# Rare missense variants of the leukocyte common antigen related receptor (LAR) display reduced activity in transcellular adhesion and synapse formation

**DOI:** 10.21203/rs.3.rs-5928514/v1

**Published:** 2025-06-02

**Authors:** Mathias Kaas, Nicolas Chofflet, Deniz Bicer, Sune Skeldal, Jinjie Duan, Benjamin Feller, Joachim Vilstrup, Rosa Groth, Suganya Sivagurunathan, Hesam Dashti, Jan Skov Pedersen, Thomas Werge, Anders D. Børglum, Beth A. Cimini, Thouis R. Jones, Melina Claussnitzer, Peder Madsen, Hideto Takahashi, Ditte Demontis, Søren Thirup, Simon Glerup

**Affiliations:** 1 Department of Biomedicine, Aarhus University, Aarhus, Denmark.; 2 The Novo Nordisk Foundation Center for Genomic Mechanisms of Disease, Broad Institute of MIT and Harvard, Cambridge, USA; 3 Synapse Development and Plasticity Research Unit, Institut de Recherches Cliniques de Montréal, Montréal, Quebec, Canada; 4 Integrated Program in Neuroscience, McGill University, Montréal, Quebec, Canada; 5 Department of Molecular Biology and Genetics, Aarhus University, Aarhus, Denmark.; 6 The Lundbeck Foundation Initiative for Integrative Psychiatric Research, iPSYCH, Copenhagen and Aarhus, Denmark.; 7 Center for Genomics and Personalized Medicine, Aarhus, Denmark; 8 Department of Medicine, Université de Montréal, Montreal, QC, Canada; 9 Imaging Platform, Broad Institute of MIT and Harvard, Cambridge, Massachusetts, USA.; 10 Department of Chemistry and Interdisciplinary Nanoscience Center (iNANO), Aarhus University, Aarhus C, Denmark; 11 Division of Experimental Medicine, McGill University, Montréal, Canada

## Abstract

The leukocyte common antigen related receptor (LAR) is a member of the LAR receptor protein tyrosine phosphatase (RPTP) family of synaptic adhesion molecules that contribute to the proper alignment and specialization of synaptic connections in the mammalian brain. LAR-RPTP members have been genetically associated with neuropsychiatric disorders, but the molecular consequences of genetic perturbations of LAR remain unstudied. Using exome sequencing data from psychiatric patients and controls, we identify rare missense variants of LAR that render the extracellular domain (ECD) unstable and susceptible to proteolytic cleavage. Using recombinant and cellular systems, we describe three variants that cause disruption of the LAR:NGL-3 interaction, which results in loss of transcellular adhesion and synaptogenic effects. Furthermore, we show that overexpression of two of these variants elicit altered morphological phenotypes in an imaging-based morphological profiling assay compared to wild type LAR, suggesting that destabilization of the LAR ECD has broad effects on LAR function. In conclusion, our study identifies three rare, missense variants in LAR that could provide insights into LAR involvement with psychiatric pathobiology.

## Introduction

Synapse formation is a highly complex process partly orchestrated by alignment of pre- and postsynaptic cell-surface adhesion molecules, of which combinatorial interactions contribute to the specification of synaptic subtypes [1–3]. One class of synaptic adhesion molecules is the type IIa receptor protein tyrosine phosphatases constituting the leukocyte common antigen related receptor (LAR), PTPδ and PTPσ, here denoted LAR-RPTPs [4, 5]. These receptors are present at synaptic terminals and axonal surfaces where they engage with postsynaptic adhesion molecules, heparan and chondroitin sulphate proteoglycans (HSPGs and CSPGs), presynaptic receptors and adaptor proteins, to properly guide axons to their targets and ensure transsynaptic tethering and function [6–10]. Like several other synaptic adhesion molecules, LAR-RPTPs have been associated to neurological and psychiatric disorders including autism spectrum disorders, Alzheimer’s Disease, attention deficit hyperactivity disorder (ADHD) and schizophrenia [11–19].

The three LAR-RPTPs share a common protein domain structure with three N-terminal Immunoglobulin-like (Ig-like) domains, up to eight fibronectin III (FN) domains, followed by a transmembrane helix and two intracellular phosphatase domains [4]. Presynaptic LAR-RPTPs interact with several postsynaptic partners across the synapse, including tropomyosin receptor kinase C (TrkC), interleukin 1 receptor accessory protein like 1 (IL1RAPL1) and the interleukin-1 receptor accessory protein (IL-1RAcP), Slit- and Trk-like receptors (Slitrks), synaptic adhesion-like molecules (SALMs) and Netrin-G ligand 3 (NGL-3) [5, 6, 20–28]. While most of these interactions are through the LAR-RPTP Ig-like domains and are regulated by mini-exon A and B (meA/B) insertions [4], interactions with NGL-3 are uniquely mapped to the FN1–2 domains [24]. In this context, LAR-RPTP:NGL-3 interactions induce excitatory synapse connections seemingly without affecting inhibitory synapses, and indeed, the FN1–2 domains of LAR-RPTPs are capable of inducing postsynaptic differentiation by themselves [24, 25].

Recent studies have demonstrated that the intracellular domains (ICDs) of LAR-RPTPs play an important role in their functions. LAR-RPTPs interact with Liprin-α(s) through their C-terminal phosphatase domain (D2), mediating lateral clustering of LAR-RPTP:Liprin-α complexes that are necessary for presynaptic differentiation [29–31]. The interaction of LAR ICDs with additional proteins such as Trio, PDGFR, catenin-β and TrkB have implicated LAR in actin remodeling, a process important for axon guidance during neuronal development [32–34].

Several of the postsynaptic and intracellular proteins interacting with LAR-RPTPs have been associated to psychiatric and neurological disorders, indicating that LAR-RPTPs participate in processes that are important for key pathways in CNS pathologies [14, 35, 36]. Furthermore, transgenic mice with perturbations of the *Ptprf*, *Ptprd* and *Ptprs* genes, encoding murine LAR, PTPδ and PTPσ, have been studied and show behavioral phenotypes such as hyperactivity, impaired spatial learning and memory, motor deficits in addition to a variety of electrophysiological phenotypes [11, 37–39].

In the present study, we used exome sequencing data from the iPSYCH cohort to map rare, missense variants to our previously reported high-resolution structures of the LAR FN1–4 domains [40]. Using recombinant protein and cellular systems we identified three damaging variants in the LAR FN1–2 domains that render LAR unable to bind its postsynaptic ligand NGL-3 and reduce synaptogenic effects *in vitro*. Using an imaging-based morphological profiling assay, we show that two of these variants elicit different morphological profiles than the WT receptor, mainly causing perturbations of actin morphology. These data indicate that disruption of the LAR ectodomain can perturb several molecular functions of LAR and potentially increase susceptibility of developing psychiatric disorders.

## Results

### LAR missense variant mapping and processing

**Figure 1:**
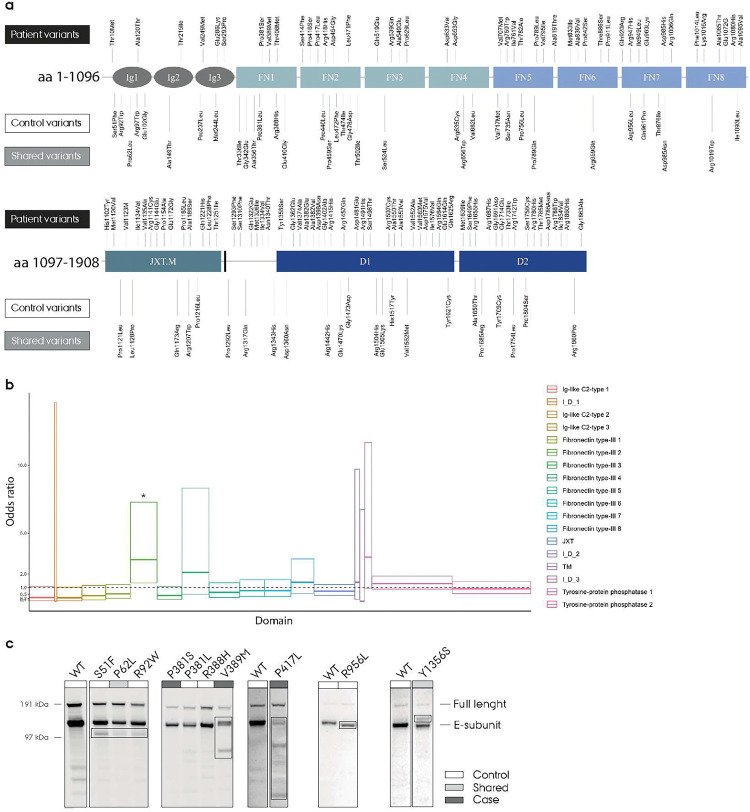
**a)** Rare missense variants identified in exome-sequencing data of individuals with psychiatric disorders and controls in the iPSYCH cohort. Variants are mapped onto a schematic illustration of the LAR receptor with the Ig1-FN8 domain on top and juxtamembraneous-D2 domain on the bottom. “Control variants” are found in individuals without any psychiatric diagnosis as defined in the iPSYCH cohorts. “Shared variants” refer to missense variants found in both patients and controls. **b)** Domain-wise burden analysis of rare, missense variants of LAR with OR and 95 % confidence interval boxes. FN2 domain shows OR=3, 95 % CI: 1.33 – 7.27, two-sided uncorrected P-value = 0.006 (Fisher exact test). **c)** Western blot analysis of selected missense variants from expression in CHO-K1 cells. Cells were left for 24 hours after transfection before lysis. Western blotting was performed with a LAR ECD specific antibody.

*PTPRF*, the gene encoding human LAR, has been associated with ADHD and schizophrenia in genome-wide association studies, but the molecular consequences of *PTPRF* perturbations have remained largely unstudied [13–15]. To investigate the effects of LAR/*PTPRF* variation, we evaluated rare missense variants in *PTPRF* (allele count ≤5 in the iPSYCH and non-Finnish Europeans from the nonpsychiatric exome subset of gnomAD [41] cohorts combined) in whole-exome sequencing data from the Danish iPSYCH cohort. These data consist of whole-exome sequences of 9,084 controls and 19,364 individuals diagnosed with at least one of the following psychiatric disorders: ADHD, autism, schizophrenia and affective disorders [42]. After QC, we identified rare missense variants at 164 sites in *PTPRF*, distributed among 183 patients and 66 controls (Fig. 1a). The increased number of missense variants observed in patients was not significant in whole-gene burden analysis (OR=1.3 (0.98 – 1.76), p=0.07, Fisher’s exact test). To evaluate whether there were domain-specific increased burden of missense variants, we supplemented the control data with rare missense variant counts from the gnomAD (non-psychiatric non-Finish European ancestry exomes subset) and found an increased burden of rare missense variants in the LAR FN2 domain in individuals with psychiatric disorders (OR=3 (1.33 – 7.27), p=0.006 Fisher’s exact test, Fig. 1b). We also identified 13 non-rare variants with high individual odds ratios (OR>3, p<0.05 Fisher’s exact test, no multiple testing correction) which were used in the following experimental studies but were not included in the burden tests described above.

To evaluate potential consequences of missense variation in LAR, we initially screened the effects of missense perturbations in a LAR processing assay. LAR is initially expressed as a single-chain receptor (approximately 200 kDa) and undergoes proteolytic cleavage of the extracellular domain upon exposure to relevant extracellular proteases, rendering E (extracellular, 150 kDa) and P (phosphatase, 85 kDa) subunits [43, 44]. We expressed LAR WT and 164 rare missense variants, as well as the 13 non-rare missense variants, in CHO-K1 cells and evaluated their processing by western blotting (Fig. S1). While a large proportion of the variants did not show any obvious processing changes, some variants showed clear differences in posttranslational processing (Fig. 1c). Here, S51F, P62L and R92W showed a band around 90 kDa not present in the WT. R956L showed a lower molecular weight of the ECD, while Y1356S showed an extra band between the usual one- and two-chain bands. Most noticeably, V389M and P417L showed different molecular weights of the ECD, and several breakdown bands were observed with lower molecular weight, suggesting that the specific amino acid changes in these variants cause destabilization and proteolytic cleavage of the ECD (Fig. 1c).

### Missense variants in the FN1–4 domain cause decreased thermal stability and altered NGL-3 binding in solution

**Figure 2:**
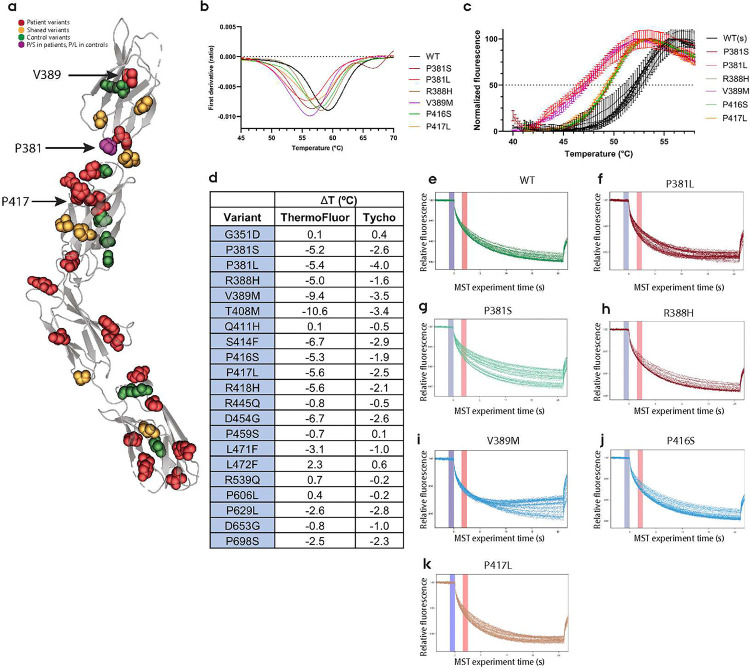
**a)** Missense variation sites on the experimental structure of LAR FN1–4 (from PDB: 6TPW [40]). Sidechains of V389 and P417 are facing the core of their respective FN domains, while P381 is facing the hinge region between the FN1–2 domains. **b)** Thermal stability of selected variants using Tycho setup. Data are represented as first-derivatives of the fluorescence 330nm/350nm ratio (n=2 pr measurement). **c)** ThermoFluor plots with Boltzmann Sigmoidal fits for selected missense variant. Horizontal dashed line indicates T_i_ (n=3 pr variant, n=3 pr WT batch using three batches for a total of n=9 replicates). **d)** Table showing alteration in melting transition point (°C change) compared to WT LAR FN1–4 for Tycho and ThermoFluor. **e-k)** MST traces for two replicates of LAR FN1–4 WT and indicated variants. Note that for P381L, V389M and P417L the traces cross during the MST-ON time (after purple vertical bar), indicative of temperature-dependent alterations.

We previously reported high-resolution structures of the LAR FN1–4 domains where the V389M and P417L variants are located [40]. Investigating the side chain positions of V389 and P417 on the experimental structures of the FN1–4 and FN1–2 domains showed that the side chains were oriented towards the center of the beta-sandwiches of FN1 and FN2 respectively (Fig. 2a and S2a-b). As side chains that are facing the inside of protein structures are often important for their structural integrity, we investigated the effects of these missense variants on protein stability using recombinant LAR FN1–4. To this end, we cloned a subset of LAR variants into a construct encoding the LAR FN1–4 domain and expressed them in E. coli as previously described [40]. We initially evaluated the stability of these variants using the Tycho NT.6 apparatus to measure the unfolding response. Here, WT LAR FN1–4 showed an inflection temperature (T_i_) of 59.2 °C. Several variants, including V389M and P417L showed a reduced T_i_, indicating that these missense variants indeed cause destabilization of the FN1–4 domain of LAR (Fig. 2b and S2d). We also observed that mutations at P381 (S/L) also caused decreased stability, although the sidechain of P381 is exposed to the hinge region between FN1 and FN2 (Fig. S2c).

To verify these findings, we customized a fluorescence based thermostability assay to LAR FN1–4 using SYPRO Orange. Here, SYPRO Orange will increase its fluorescent signal when exposed to the hydrophobic cores of proteins whilst they unfold [45]. We screened the unfolding response of FN1–4 missense variants to confirm the data seen from the Tycho assay. This assay revealed a similar pattern of changes in unfolding response of the LAR FN1–4 mutants, although with a lower baseline T_i_ for the WT, but also higher resolution for the changes caused by missense mutations (Fig. 2c–d, S2e). In general, variants in the FN1 domain and the small hinge region between the FN1 and FN2 domains had a stronger effect on thermostability compared to variants in the other three FN domains. Again, the P381L/S, V389M and P417L variants were demonstrated to have marked effects and caused reduction of the thermal stability of the LAR FN1–4 domain. The T_i_ for these variants was significantly different from WT when comparing the V50 values from the Boltzmann sigmoidal fit (p<0.001).

As the FN1–2 domains of LAR are solely responsible for the LAR:NGL-3 interaction [25], we investigated whether missense variants in this region would affect LAR:NGL-3 binding properties. To this end, we set up a microscale thermophoresis (MST) assay to assess LAR FN1–4 binding to the NGL-3 ECD. Measurements of the affinity constant for the WT LAR:NGL-3 interaction were found to have a K_D_ of 3.68 μM (Fig. S2f). When testing the P381L, V389M and P417L variants, we found distinct MST patterns differing from the WT, indicative of a temperature dependent alteration of the LAR:NGL-3 interaction (Fig. 2f,i,k). The P381S variant showed a higher affinity for NGL-3 than LAR WT (Fig. 2g), but as this variant showed NGL-3 dependent differences in initial fluorescence (not shown), the K_D_ should be interpreted with caution. The remaining variants tested did not show notable changes in affinity nor MST trace patterns (Fig. 2h,j, S2g and data not shown).

Taken together, these data suggests that rare missense variants found in the FN1–2 domain of LAR alter the stability of these domains and, for three specific variants, potentially cause disruption of the interaction with its postsynaptic ligand NGL-3.

### LAR variants P381L, V389M and P417L are unable to induce transcellular adhesion

**Figure 3:**
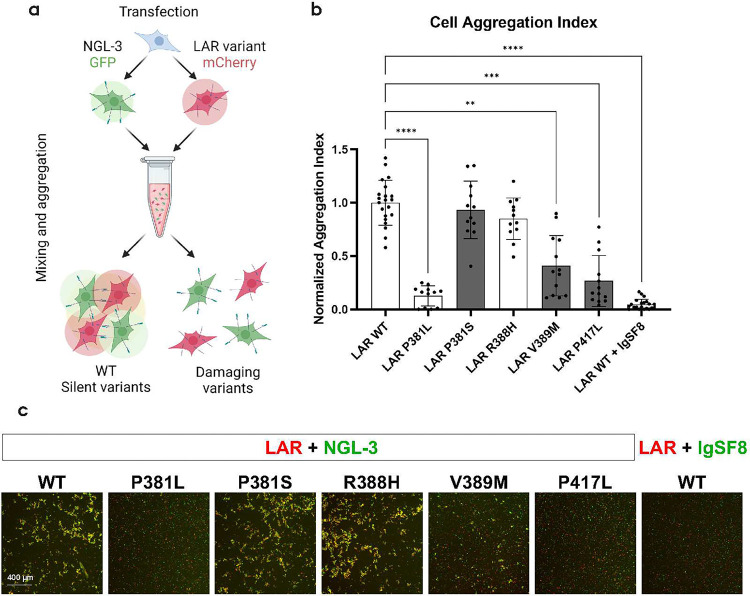
**a)** Illustration of the transcellular aggregation assay using LAR and NGL-3. Here, separate wells of HEK293T cells are co-transfected with NGL-3 + GFP or LAR + mCherry and subsequently resuspended and mixed in solution and then analyzed with fluorescence microscopy to evaluate amount of aggregated cells **b)** Quantification of cell aggregation index (area) for LAR WT and selected variants as well as IgSF8 (negative control). Bar coloring indicates case-control status (control variants: white, case variants: dark grey). The experiment constitutes at least three biological replicates of n=4 images (12 in total) pr variant. **p=0.0047, ***p=0.0001, ****p<0.0001 (Kruskal-Wallis test). Data is presented as mean ± SD. **c)** Representative images from fluorescent microscopy of cell aggregation solutions as described in a.

To gain a better understanding of the functional consequences of the variants in the FN1–2 domains of LAR, we evaluated the effects of the identified missense variants on NGL-3 binding in cellular systems. A primary function of LAR-RPTPs is to induce transcellular adhesion by interacting with postsynaptic ligands [4]. This can be assessed in non-neuronal systems, as cells (over)expressing LAR-RPTPs will aggregate with cells expressing their postsynaptic ligands [24, 46]. As the data from recombinant FN1–4 variants in cell-free assays indicated that several variants, including P381L, V389M and P417L, changed the structural stability of the FN1–2 domains known to interact with NGL-3, we explored whether these variants caused perturbations of LAR:NGL-3 transcellular interactions by the cellular aggregation assay [24, 25]. Accordingly, we co-transfected HEK293 cells with LAR variants and mCherry or NGL-3 and GFP and mixed the cells in suspension 24 hours post transfection followed by imaging analysis (Fig. 3a). In agreement with previous studies, WT LAR and NGL-3 readily induced transcellular adhesion, but introduction of variants P381L, V389M or P417L resulted in >50 % mean, significant decrease in cellular aggregation (Fig. 3b–c). We also assessed 19 other variants throughout the LAR protein to determine whether this effect was specific for P381L, V389M and P417L. Although only a subset of missense variants was tested, none showed significant differences from WT LAR (Fig. S3a-b), suggesting that the observed loss of adhesion is specific to P381L, V389M and P417L. Notably, T408M, which was as unstable as V389M in the stability assays, and P381S which had apparent higher affinity for NGL-3 in MST, did not differ from LAR WT in this assay.

### Loss of NGL-3 binding renders LAR variants unable to induce synapse formation

**Figure 4:**
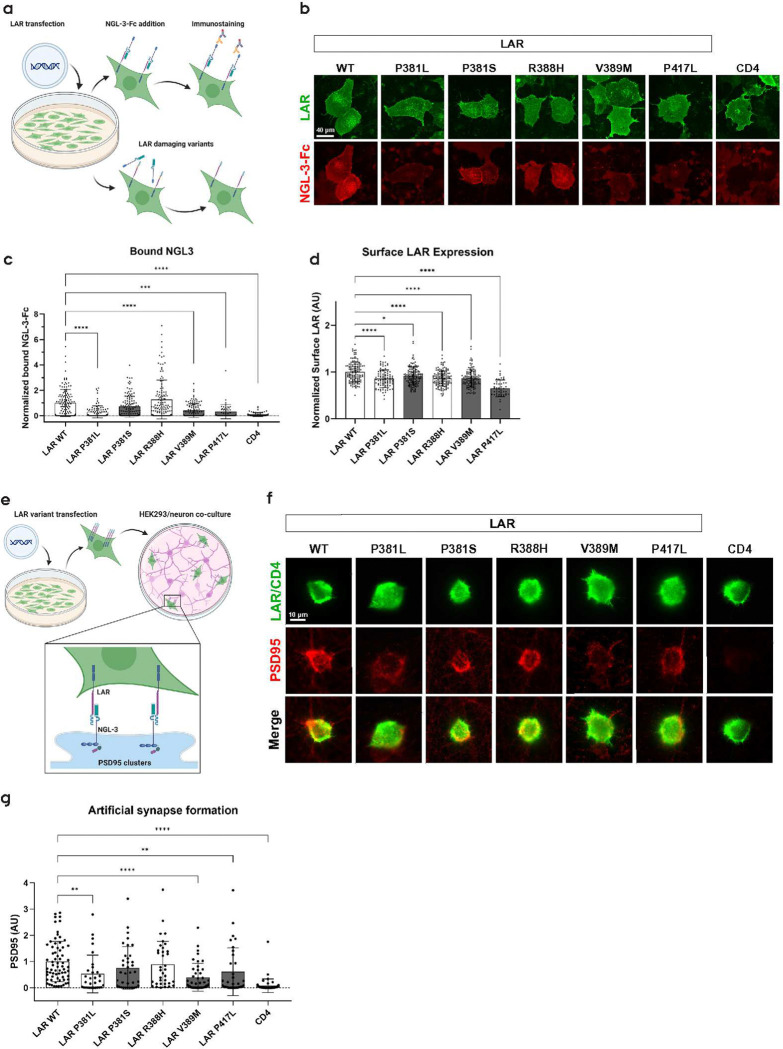
**a)** Illustration of *in situ* binding assay using LAR variant transfected HEK293T cells and Fc-NGL-3 fusion protein. Cells were transfected on coverslips and exposed to 2.5 μM soluble Fc-NGL-3 and immunostained (without permeabilization) to visualize the amount of Fc-NGL-3 binding. **b)** Representative fluorescent images of LAR transfected cells with surface-bound NGL-3. **c)** Quantification of surface NGL-3 binding for LAR WT and selected variants, as well as CD4 as negative control binder for NGL-3 (at least three biological replicates and n=49 cells pr replicate. ***p=0.002, ****p<0.0001 (Kruskal Wallis test). Data are presented as mean ± SD. **d)** LAR Surface expression quantification from NGL-3 *in situ* binding assay. All variants showed less signal at the cell surface compared to WT. *p=0.0141, ****p<0.0001 (Kruskal Wallis test). **e)** Illustration of artificial synaptogenesis assay using LAR transfected HEK293T cells seeded onto rat hippocampal neurons. The cells were co-cultured for 24 hours before PSD-95 assessment. **f)** Representative images of LAR transfected HEK293T cells in co-culture with hippocampal neurons stained for PSD-95 clustering. **g)** Quantification of PSD-95 clusters as markers of excitatory synapse formation onto LAR transfected HEK293T cells, using CD4 as a negative control. Data consists of three biological replicates with n>10 images pr replicate. **p<0.005, ****p<0.001 (Kruskal Wallis test). Data are presented as mean ± SD. Bar coloring in c), d) and g) indicates case-control status (control variants: white, case variants: dark grey)

To determine whether the loss of transcellular aggregation was a result of loss of NGL-3 interaction *in vitro*, we tested the binding of full-length LAR variants to NGL-3 at the cell surface. We transfected COS7 cells with WT LAR or P381L, V389M and P417L variants, and subjected the cells to a soluble NGL-3-Fc fusion protein (Fig. 4a). NGL-3-Fc was detected at significantly lower levels at the surface of COS7 cells transfected with variants P381L, V389M and P417L compared to WT control and variants P381S and R388H (Fig. 4b–c). Quantifications were normalized to the surface expression of LAR, where all tested variants showed significantly reduced surface expression (Fig. 4d).

As LAR is capable of inducing excitatory postsynaptic differentiation through NGL-3 and other postsynaptic ligands [24, 25, 47], we explored whether the loss of transcellular adhesion and NGL-3 interaction shown by the P381L, V389M and P417L variants translated to loss of postsynaptic induction in an artificial synapse formation assay. Here, we transfected HEK293 cells with LAR WT or missense variants and seeded them onto DIV14 rat hippocampal neurons. This mixed culture was PFA fixed 24 hours after HEK293 cell seeding and immunostained for PSD-95 (Fig. 4e). HEK293 cells expressing LAR WT readily induced postsynaptic differentiation from the co-cultured neurons as demonstrated by increased PSD-95 signal in proximity to these cells, which was not observed for the negative controls transfected with CD4 (Fig. 4f–g). In line with the loss of transcellular adhesion, the P381L, V389M and P417L missense variants displayed a significant decrease in postsynaptic induction, while variants P381S and R388H showed no difference in accumulation of PSD-95 clustering when compared to WT LAR (Fig. 4f–g). In this experiments, we selected HEK293 cells with similar surface expression of each LAR construct in order to assess the effect of the reduction of LAR:NGL3 trans-interaction and not LAR surface expression itself (Fig. S4a).

Since the full-length LAR constructs used so far do not contain meA or meB inserts, it is unlikely that they could induce postsynaptic differentiation through their Ig-like domains, since the majority of known postsynaptic adhesion partners through these domains depend on combinatorial meA/B insertions [22, 48]. Thus, directly interpreting whether the loss of synaptogenic effects, seen with P381L, V389M and P417L, is caused by loss of NGL-3 binding or FN1–2 domain destabilization (and potential breakdown of the N-terminal part of the ECD) is difficult. To evaluate if these variants retained functional integrity of the N-terminal Ig-like domains, we tested their ability to bind postsynaptic partners through these domains when expressing the meA and meB insertions. In a similar cell surface binding assay as performed above, we tested LAR^meA+/meB+^ variants binding to SALM5-Fc [26]. Here, P417L was the only variant that showed decreased binding to SALM5-Fc (Fig. S4b+c). Furthermore, P417L was also the only variant that showed lower surface exposure among the variants (Fig. S4d).

### The LAR FN1–4 domains are disrupted by variants P381L, V389M and P417L

**Figure 5:**
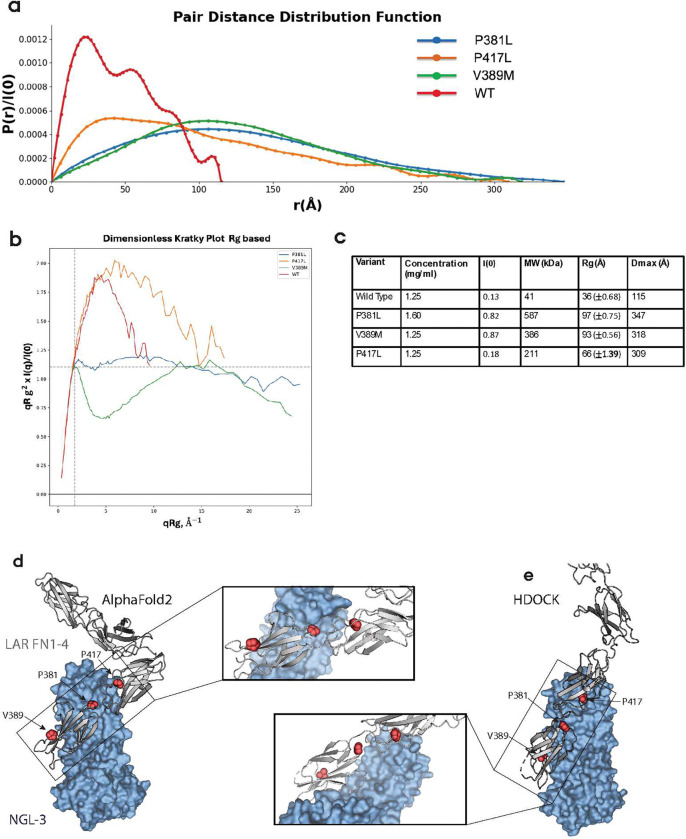
**a)** Pair distance distribution function (indirect Fourier transformation [IFT] of SAXS data) of LAR variants for analysis of distance distribution histogram to estimate the shape of protein, whoing elongated plots for the three tested variants indicating elongated protein shapes. **b)** Dimensionless Kratky plot based on Radius of gyration (Rg) of LAR variants for compactness and folding state analysis. **c)** Structural parameters of LAR rare variants calculated from SAXS data based on Guinier analysis and IFT. I (0), forward scattering intensity, MW, molecular weight estimate, Rg(Å) Radius of gyration, and Dmax(Å), maximum particle dimension. **d)** Structural modelling of LAR FN 1–4 and NGL-3 interaction using AlphaFold2 and **e)** Molecular docking program HDOCK.

As cellular processing of full-length LAR, as well as thermal stability and MST analysis of the (recombinant) FN1–4 domains indicated destabilization by the P381L, V389M and P417L variants, we speculated whether these variants caused unfolding of the FN1–2 domains which then in turn affected the interaction with NGL-3. To test if the P381L, V389M and P417L variants had generalized effects on the structure of the FN1–2 domains, we used small-angle x-ray scattering (SAXS) analysis for the recombinant FN1–4 variants, as we had previously used this method to determine solution structures of the WT LAR FN1–4 domain [40]. The P381L, V389M and P417 variants all showed large differences in scattering profiles (log(I(q)) versus log(q)) and pair-distance distribution functions (PDDF, a histogram of distances between pair of points weighted by the excess scattering length in the points) compared to WT LAR FN1–4, indicating altered protein structure where the PDDF suggests asymmetric and/or elongated structures of the variants (Fig. 5a, S5a). Moreover, dimensionless Kratky analysis showed that variant P381L was highly flexible and probably denature easily (Fig. 5b). Guinier fit analyses are shown in Fig S5b-e and other SAXS statistics in figure 5c.

As no experimental structures are available for the LAR:NGL-3 complex, we used AlphaFold2 [49] and molecular docking simulations to model the LAR FN1–4:NGL-3 interaction to estimate the position of P381, V389 and P417 in relation to NGL-3. Here, both AlphaFold2 (available through ColabFold [50]) and HDOCK simulations [51], using the sequences or published (individual) structures of LAR FN1–4 domains and NGL-3 ECD domains respectively, suggested that the FN1–2 domains of LAR interacts with the concave surface of the NGL-3 LRR domain (Fig. 5d–e, S6a-b). The interaction involved beta-sheet surfaces of FN1 as well as the FN1–2 hinge region and N-terminal FN2 loops (Fig. 5d–e and S6a-b). AlphaFold and HDOCK, however, predicted opposite beta-sandwich surfaces of the FN1 domain to be involved. In the AlphaFold model, the P381L variant was seemingly in the interaction surface of LAR:NGL-3, whereas the V389M and P417L variants were placed too far from NGL-3 to be directly involved in the interaction (Fig. 5d). Neither of the variants were found to be directly involved in the LAR:NGL-3 interaction in the HDOCK model (Fig. 5e). Both models were high-confidence predictions as illustrated by PAE plots for AlphaFold (Fig. S6c) and HDOCK docking score of −295.67 and confidence score 0.9485.

Taken together, SAXS and computational modeling suggest that P381L, V389M and P417L potentially cause unfolding of the FN1–2 domains to limit interactions with NGL-3.

### Imaging-based morphological profiling illustrates broad effects of damaging LAR missense variants

**Figure 6:**
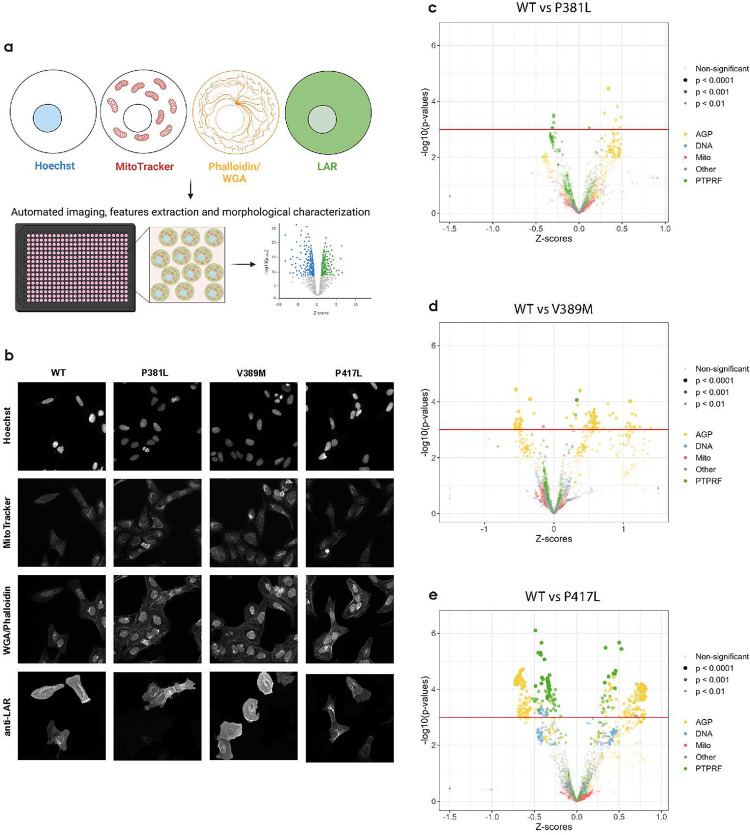
**a)** Graphical illustration of adapted LP assay to evaluate morphological features elicited by LAR WT or variant overexpression in U2OS cells. Cells were multi-stained with Hoechst, MitoTracker, Phalloidin, WGA and anti-LAR in 384 well plates. Images were acquired with automated, high-content confocal microscopy and feature profiles were extracted for single cells and aggregated for each well before undergoing customized analysis. **b)** Representative images of the four channels from WT, P381L, V389M and P417L illustrating staining of specific compartments and identification of LAR-positive cells. **c-e)** Volcano plots illustrating changed feature types between WT LAR and P381L, V389M and P417L respectively. Dot size is scaled dependent on the p-value as illustrated. Data is from n=5–6 wells with a minimum of 20 LAR-positive cells pr well. p-value from t-test. X-axis are clipped at −1.5 and 1.5.

LAR-RPTPs are involved in several cellular processes other than synapse formation including neurite outgrowth, dephosphorylation of intracellular targets, actin remodeling, filopodia formation and cell migration [32, 33, 52, 53]. Thus, damaging LAR missense variation might have effects beyond those evaluated in classical adhesion and synaptogenic assays. In an attempt to evaluate whether LAR missense variation could have broader molecular effects, we adapted the recently published LipocyteProfiler assay (itself an adaption of the Cell Painting assay [54, 55]), that utilizes high-content confocal imaging and computational feature extraction to capture thousands of morphological features from single cells and systematically interrogate genetic or chemical perturbations [56, 57]. We transfected U2OS cells with LAR WT or the P381S/L, R388H, V389M, P416S and P417L variants, and after 24 hours we performed an adapted Lipocyte Profiler staining protocol, using MitoTracker, Wheat Germ Agglutinin (WGA), Phalloidin, Hoechst and an α-LAR antibody, the latter replacing the SYTO-14 and BODIPY stains. Using automated confocal imaging, we captured images of U2OS cells and performed a single cell analysis pipeline that identified transfected cells based on the α-LAR signal and extracted ~3000 features per cell across five imaging channels. Using pr well mean readouts for each feature, we calculated per-feature Z-scores and p-values for each LAR variant compared to WT (Fig. 6a–b).

When comparing WT LAR with the variants P381L, V389M or P417L, there was a clear change in morphological feature characteristics for V389M and P417L (Fig. 6c–e). Most notably different from LAR WT was P417L with 329 features significantly changed from the WT, while V389M had 112 features and P381L had only 13 significant changes (p<0.001, t-test, Fig. 6c–e). Common for V389M and P417L comparisons with WT were a high proportion of Actin-Golgi-Plasma membrane (AGP) features altered between the groups, of which most were related to cytoplasmic texture features (Supplementary Table 1). Analysis of LAR WT against P381S, R388H and P416S showed only 4, 5 and 1 features significantly changed, suggesting that these variants are generally well tolerated and do not induce large changes to LAR function (Fig. S6a-c). Overall, the imaging-based profiling suggested that the V389M and P417L LAR variants perturb functions of LAR not specific for synapse formation.

## Discussion

In the present study, we used rare missense variants identified in individuals diagnosed with psychiatric disorders and controls to investigate the biological effects that might underlie the genetic association of *PTPRF*/LAR with psychiatric disorders. We identified three rare missense variants in the LAR FN1–2 domains that caused loss of synaptogenic and adhesion properties of LAR. Among these variants, P381L was only found in controls, while V389M and P417L, that also elicited more general perturbations of LAR function, were found in patients only.

Studies of full-length LAR processing, SAXS and the adapted LP assay suggest that destabilization of the FN1–2 domains by V389M and P417L may result in LAR instability and/or accelerated turnover. This could result in increased loss of (fragments of) the LAR ECD, which in turn could also cause general loss of synaptic adhesion through the LAR extracellular domain. Cell surface shedding of synaptic adhesion molecules has been described as a key regulatory mechanism for their activity and function [58], and soluble LAR-RPTP ectodomains have been shown to regulate neurite growth and response to nerve injury [59–63]. Furthermore, the excess soluble LAR ectodomain fragments at synaptic terminals could act as decoy receptors for postsynaptic LAR-RPTP ligands to prevent transsynaptic complex formation, as proposed for other synaptic adhesion molecules [58]. During development, excess soluble LAR ectodomain could act as guidance cues for neighboring neurons during development while the LAR expressing neurons would lack membrane anchored LAR to participate in axon guidance, providing potential mechanisms by which the identified variants could participate in psychiatric pathogenesis.

The loss of synaptogenic potential of the LAR P381L, V389M and P417L variants shown in this study is plausibly explained by loss of NGL-3 binding, since the same variants expressed in a LAR construct containing meA and B inserts could bind the postsynaptic ligand SALM5. Thus, LAR could potentially still induce synapse formation, through its Ig-like domains, in the absence of NGL-3 binding. MeA and B insertions in the Ig-like domains are important for the majority of interactions of the Ig-like domains of LAR-RPTPs, and LAR-RPTPs devoid of both these inserts generally have low affinity for postsynaptic ligands interacting with their Ig-like domains [5]. A recent study has thoroughly characterized the presence of mini-exon inserts in LAR-RPTPs throughout several brain regions, neuronal subtypes and projections in mice [48]. Specifically, they showed that the majority of excitatory neurons in the cortex and thalamus are devoid of the meA and B inserts, suggesting the importance of LAR:NGL-3 binding in specific circuits. Thus, the NGL-3 specific synaptic perturbations caused by the P381L, V389M and P417L LAR variants could be highly context or spatially dependent *in vivo*.

Lateral clustering of LAR-RPTPs is critical for synaptogenesis and actin remodeling and is mediated by both intracellular adaptor molecules, such as Liprin-αs, and extracellular ligands [30, 33, 64, 65]. The LAR D1 domain has phosphatase activity which is inhibited after Liprin-α induced clustering, suggesting that LAR-RPTP signaling is regulated by receptor dimerization [31]. In addition, the LAR-RPTP ICD undergoes redistribution after shedding of the ectodomain, indicating that release of the ECD could have consequences for downstream intracellular signaling of LAR [66]. While we did not investigate the distribution or processing of the LAR ICD in the present work, the changed morphological features elicited by expression of the V389M and P417L variants could be a manifestation of altered LAR clustering and/or intracellular signaling caused by destabilization of the LAR ectodomain. Additionally, the ECDs of LAR-RPTPs are well described to interact with extracellular matrix proteins such as laminin and syndecans and other HSPG/CSPGs to modulate focal adhesions and actin morphology [53, 67–70], and distortion of such extracellular interactions through ectodomain destabilization could in turn perturb LAR clustering and downstream signaling. As such, the altered AGP morphology observed using imaging-based morphological profiling could be caused by dysfunctional regulation of the LAR ICD due to ectodomain destabilization. Thus, the LAR V389M and P417L variants could potentially contribute to psychiatric pathobiology through several mechanisms *in vivo*, in line with the observed effects in the adapted LP assay. Indeed, unbiased morphological phenotyping represents a more powerful approach to evaluate the effects of a large number of missense variants, and can potentially capture both known and unknown functions of the proteins and/or variants in question [71].

The three highlighted variants are rare, and in the present study we lacked statistical power to argue any genetic association with psychiatric disorders, and indeed, P381L was only found in controls. The understanding of psychiatric genetics has improved over the last decade due to large GWAS and rare variant studies and have demonstrated that psychiatric disorders have a highly heterogenous genetic makeup with contributions from thousands of both rare and common variants [14, 15, 72]. The presence of damaging, rare variants on top of a high polygenic risk score for psychiatric disorders contribute significantly to psychiatric risk, but by themselves, these variants are not necessarily causing psychiatric disorders [14, 73, 74]. In addition, data from gnomAD suggests that *PTPRF* is evolutionarily constrained as indicated by LOEUF and missense Z scores of 0.35 and 4.05 respectively [75], and as such, the damaging phenotypes elicited by P381L, V389M and P417L could be due to general missense intolerance of *PTPRF* rather than specific association with psychiatric disorders. However, the findings in the present study argue for further attention to missense variation in LAR-RPTPs and their potential association with psychiatric disorders.

Taken together, we present the identification of three rare missense variants in the LAR FN1–2 domains that perturb transcellular adhesion, synapse formation and broader LAR functions *in vitro* and propose that such damaging missense variation in LAR could contribute to psychiatric pathobiology.

## Materials and methods

### Whole Exome Sequencing analysis

In our study, we utilized the Case-Cohort dataset, iPSYCH2012 [42], established by the Integrative Psychiatric Research (iPSYCH) consortium, rooted in the Danish demographic. The dataset comprises all samples linked to a psychiatric diagnosis (as specified below) and 30.000 randomly chosen population-based controls. A subset of the iPSYCH2012 dataset, spanning data from 34,544 individuals, presents whole exome sequencing (WES) data of both control participants and those diagnosed with any of the five salient psychiatric maladies as logged in the Danish Psychiatric Central Research Register by the end of 2015. These disorders encompass attention-deficit/hyperactivity disorder (ICD10: F90.0), autism spectrum disorder (ICD10: F84.0, F84.1, F84.5, F84.8 or F84.9), bipolar disorder (ICD10: F30-F31), schizophrenia spectrum (ICD10: F20-F29), and affective disorder (ICD10: F30-F39).

WES analyses were performed on DNA extracted from dried blood spots; a method that has previously been utilized for generating high-quality WES data [76]. After DNA extraction the samples were whole-genome-amplified in triplicate as previously described [77]. Using the Illumina Nextera capture kit the coding regions were isolated and subsequently sequenced on an Illumina HiSeq platform at the Genomics Platform at the Broad Institute of MIT and Harvard, Boston, MA. Raw sequencing data was aligned to the reference genome (Hg19). Genotype-calling was performed as suggested by Genome Analysis Toolkit v.3.4. and QC steps were performed with Hail 0.1 (Hail Team, https://github.com/hail-is/hail). Variants were only included in the analysis if they were located in high-confidence regions in both the iPSYCH and GnomAD databases [41] defined as >80% of the samples having more than 10 x coverage in the genomic region in question. After these QC steps, the dataset was streamlined to represent 28,448 participants, which includes 19,364 cases and 9,084 controls, and a set of 1,352,490 high-quality genetic variants. Annotation of these variants was achieved through SnpEff [78] version 4.3t. Additional allele count details from the gnomAD [41] exomes r2.1.1 database were annotated via SnpSift [79] version 4.3t. Within the scope of our research, we classified variants as ‘rare’ if their allele count was no greater than 5 across our iPSYCH cohort (n=28,448) and the subset of non-psych non-Finnish European of gnomAD (n=44,779).

### Mapping of rare missense variants and bioinformatical analysis

From the WES data subset of iPSYCH2012 we extracted all variants of *PTPRF* within the gene positions chromosome 1 43991708–44089343. The genomic positions and annotated nucleotide changes were subjected to the Variant Effect Predictor (VEP) [80] tool, using the GRCh37.p13 assembly. As *PTPRF* has many transcripts and thus for most (coding) variants VEP presents >5 consequences pr mutation, the longest transcript of *PTPRF* was selected as the reference, and this transcript represents the canonical Uniprot sequence (ID: P10586). To gain more power for the subsequent domain-wise analysis, we incorporated variant counts from the non-neuro non-Finnish gnomAD cohort into the analysis as well. Whole-gene and per domain burden tests were done with Fisher’s Exact test.

### Plasmid preparation

The human *PTPRF* construct was purchased from Genscript inserted into a pcDNA3.1/Zeo(+) vector. This parent plasmid was exposed to high-throughput site-directed mutagenesis as offered by Genscript to create 164 PTPRF rare and 13 non-rare missense variants for cellular experiments.

Human *PTPRF* construct encoding the FN1–4 domains was purchased from Genscript inserted into a pET-9a vector as previously described [40]. The plasmids contained N-terminal 6xhis tag bridged by a TEV cleavage site. This construct was also subjected to mutagenesis to obtain missense variants of the FN1–4 domain.

Plasmid for Fc-NGL-3 was produced as previously described [81]. In brief, the annotated sequence for the extracellular domain has been inserted in frame using 5’ Notl and 3’ Xbal sites in a modified pCMV6-XL4 vector. This vector inserts the coding region between a C-terminally fused Fc protein bridged by a 3CPro cleavage site (LEVLFQ/GP) and N-terminally prolactin leader peptide (MDSKGSSQKGSRLLLLLVVSNLLLCQGVVSTPVV) and Flag-tag (DYKDDDDK).

For use in cell-surface binding, cellular aggregation and artificial synapse formation assays, the following constructs have previously been described: extracellularly HA-tagged human CD4 (HA-CD4) [82], intracellularly CFP-tagged rat IgSF8 (IgSF8-CFP) [83] and NGL-3 (NGL-3-CFP) [84]. The plasmid pCAGGS-mCherry was a gift from Phil Sharp (Addgene plasmid # 41583) [85].The plasmid pCAG-GFP was a gift from Connie Cepko (Addgene plasmid # 11150) [86].

### Cell culturing and rare variant processing analysis

CHO-K1 cells were maintained at 37 °C and 5 % CO_2_ in either SFM Hybridoma (cat) or F-12 (cat) media supplemented with 10% FBS. For full length LAR variant analysis, CHO-K1 cells were seeded in 24 well plates at ~100K cells/well. 24 hours after seeding, medium was replaced with SFM Hybridoma media with no additives. Cells were transfected with LAR variants with FuGENE (Promega), using 256 ng DNA and 1 μL FuGENE pr well, and left for 24 hours before the medium was harvested and cells were lysed in 100 μL TNE lysis buffer with cOmplete and PhosStop for 20 minutes on ice. Cells were scraped off and transferred to 1.5 mL Eppendorf tubes and spun at 4500 rpm for 10 minutes at 4 °C before being used for western blotting.

### SDS-PAGE and Western blotting

Cell lysates were spun down at 4500 rpm at 4°C for 10 minutes and cell pellets were discarded. 10–20 μL of lysate was mixed with ¼ of total volume LDS Sample Buffer (Invitrogen, #NP0007) and 1/10 of total volume DTT. The protein mixture was heated to 95 °C for 5 minutes before being loaded on a 4–12 % Bis-Tris gel (Invitrogen) in NuPAGE MOPS buffer (ThermoFisher, #NP0001)). The proteins were transferred to nitrocellulose membrane using iBlot2 Transfer Stack kit (Invitrogen, NB301001) and iBlot2 Dry Blot System (Invitrogen, #IB1001). The membrane was blocked in 50mM Tris-base, 500mM NaCl, 2% Milk powder and 2% Tween-20) for at least 30 minutes and incubated with primary LAR antibody (R&D systems, AF3004) recognizing the LAR ECD overnight (ON). The antibodies were diluted in blocking buffer. The next day, the membrane was washed three times for 10 minutes in washing buffer (CaCl2 2mM, MgCl2 1mM, HEPES 10mM, NaCl 140mM, 0.2% Milk powder and 0.5% Tween-20) and incubated with HRP-conjugated secondary antibody (diluted 1:2000 in blocking buffer, rabbit α-goat (DAKO #0160) for 1 hour at room temperature. Next, the membrane was washed 3×5 min in washing buffer and bands developed using Amersham ECL Western Blotting Detection Reagent (GE Healthcare, # RPN2106) or SuperSignal West Femto Maximum Sensitivity Substrate (ThermoFisher Scientific, #34096) - following supplier’s instructions - and detected and analyzed using LAS-4000 and appurtenant software (GE Healthcare) or iBright^™^ CL1500 Imaging System (A44114, Invitrogen). The intensity of bands was quantified by densitometric analysis using Multi Gauge V3.2 software.

### Protein production and purification

The production of LAR FN1–4 constructs was performed as previously described [40]. The plasmids for FN1–4 WT or mutants were transformed into E. coli BL21(DE3) cells. Transformed cells were cultured at 37°C in LB broth medium containing 100 μg/mL kanamycin. When optical density reached 0.85 at 600 nm the culture was supplied with 1 mM isopropyl B-D-1-thiogalactopyranoside (IPTG) to induce protein expression and the culturing temperature was lowered to 30°C and left ON for 14–16 hours at 225 rpm. The cells were harvested by centrifugation at 6000g for 20 minutes at 4°C and pellets were stored at −20°C. The cells were lysed in by sonication pulses of 30 % for 3 × 5 minutes on ice. Bacterial debris was removed by ultracentrifugation at 10,000g for × min at 4°C. His-tagged protein was extracted by either application to a HisTrap HP column (GE Healthcare) at 1 ml/min or by application onto Talon beads. In both cases, the column/beads were washed in 50 mM Tris pH 7.4, 300 mM NaCl, 20 mM imidazole, and the protein eluted in elution buffer containing 50 mM Tris pH 7.4, 300 mM NaCl, 400 mM imidazole. Eluted protein was dialyzed overnight at 4 °C against PBS pH 7.5 containing 10 % glycerol The dialyzed protein solution was exposed to size-exclusion chromatography (SEC) using a Superdex 200 Increase 16/600 column (GE Healthcare) after equilibration to a SEC buffer containing 50 mM HEPES pH 7.5, 50 mM NaCl. Peak SEC fractions were analysed with SDS-PAGE and upon confirmation of protein size were pooled and concentrated to ~3 mg/mL before storage at −80°C.

Production of Fc-fusion proteins was performed using the ExpiCHO system (Gibco A29133). ExpiCHO-S^™^ cells were maintained in suspension in 125-mL polycarbonate Erlenmeyer shaker flasks at 37 °C, >80% humidity and 8 % CO_2_ at ~125 rpm using ExpiCHO^™^ Expression Medium. One day before transfection, cells were seeded at 4*10^6 cells/mL in 120 mL Expression Medium. On the day of transfection, cells were diluted to a final concentration of 6*10^6 cells/mL. 1 μg of DNA pr mL of culture was diluted in ice-cold OptiPRO medium and mixed with ice-cold ExpiFectamine-OptiPRO mixture and left at RT for 5 minutes before adding to the shaker flask containing the ExpiCHO-S cells. 18–22 hours post transfection, the cultures were supplemented with freshly prepared mixture of ExpiFectamine CHO Enhancer and ExpiCHO Feed. Protein was harvested at 8–10 days after transfection by centrifugation at 6000g for 10 min at 4°C. For purification, the solution was subjected to a Protein A column at 1 mL min^−1^ at 4 °C ON. The Fc-region was cleaved off utilizing the 3C cleavage site with 3C protease. The protein solution was exposed to size-exclusion chromatography (SEC) using a Superdex 200 Increase 16/600 column (GE Healthcare) after equilibration to a SEC buffer containing 50 mM HEPES pH 7.5, 50 mM NaCl. Peak SEC fractions were analysed with SDS-PAGE and pooled and concentrated to ~3 mg/mL and stored in 50 mM HEPES pH 7.4, 50 mM NaCl at −80 °C.

### Thermostability using Tycho NT.6

Alterations to LAR FN1–4 stability were assessed using the Tycho NT.6 apparatus from NanoTemper. LAR FN1–4 variant samples were prepared at ~25 μg/mL in 50 mM HEPES pH 7.5, 50 mM NaCl. The samples were subjected to a temperature increment from 30–95 °C and the intrinsic fluorescence of tryptophane and tyrosine aromatic rings at 330 nm and 350 nm were measured throughout. The ratio of 330/350 nm fluorescence was used to analyze the thermal transition temperatures. Inflection temperatures were calculated automatically with the inherent Tycho NT.6 software.

#### ThermoFlour assay:

Screening of melting transition point of LAR FN1–4 mutants was performed using protein mixtures with SYPRO Orange. A mixture of 4 μM LAR FN1–4 protein and 5 x SYPRO orange was diluted in 50 mM HEPES pH 7.5 50 mM NaCl and loaded into 96-well qPCR plates in triplicates. Several LAR FN1–4 WT batches were spread across the plate to eliminate positioning effects. Melting transition was assessed using an AriaMx Real-time PCR System (Agilent) with FAM filter. The AriaMx was programmed to perform a melting curve with a temperature curve from 40–95 °C in 0.2 °C increments, with each increment lasting 5 seconds. Intensity signals were measured with the SYBR/FAM filter. Raw intensities were normalized to the highest value and values above 60 °C were omitted from the analysis. Melting transition points were analyzed by Boltzmann sigmoidal fits in Graphpad Prism.

### Microscale Thermophoresis

LAR FN1–4 variants were analyzed for binding to NGL-3 using Microscale thermophoresis (MST). His-tagged LAR FN1–4 WT and mutants were labelled with RED-tris-NTA 2^nd^ generation dye (MO-L018, NanoTemper). Optimal labeling conditions were determined as 200 nM LAR FN1–4 and 25 nM fluorophore in 25 mM HEPES pH 7.5, 150 mM NaCl, 0.01 % Tween20 with incubation for 20 min at RT followed by centrifugation for 10 min at 15000g at 4 °C. 1:1 dilution series of NGL-3 were prepared in 25 mM HEPES pH 7.5, 150 mM NaCl in PCR tubes and labeled LAR FN1–4 was added to a final concentration of 100 nM and 0.005 % Tween20. Samples were briefly spun down and incubated at RT for 1 h protected from light. Samples were then loaded into MonolithTM NT.115 Monolith Premium Capillary Chips and run on a Monolith NT.115 (NanoTemper Technologies GmbH). MST was run with 100 % LED power, medium MST power with 3 seconds initial fluorescence, 20 seconds MST on time and 1 second back diffusion. The data was acquired with MO.Control software and analyzed using MO.Affinity Analysis (NanoTemper Technologies GmbH). MST on time for analysis was set to 2.5 s and the data was fitted using a 1:1 stoichiometry K_D_ model inherent to the software.

### Cellular aggregation assays

Two groups of HEK293T cells grown in 12-well plates were transfected with the appropriate plasmids using TransIT-LT1 (Mirus Bio. LLC; #MIR2305) and cultured in Dulbecco’s modified Eagle’s medium (DMEM) (Gibco; #11965118) containing 10% (v/v) fetal bovine serum (FBS) (Wisent; #080–150). After 48 hours, HEK293T cells were trypsinized and resuspended in 250 μl of DMEM containing 10% (v/v) FBS before mixing of appropriate GFP- and mCherry-expressing cells to a 1:1 ratio. Mixed HEK293T groups were rotated at room temperature for 2 hours to allow cells to aggregate. Cell mixtures (500 μl) were added to 24-well plates and then imaged on an IncuCyte S3 Live-Cell Analysis System (Sartorius) with a 4x air objective. Images were acquired as 16-bit grayscale.

### *In situ* surface binding assays using soluble proteins

Protein binding assays were performed as described previously [87, 88]. Briefly, to assess protein binding on the surface of COS7 cells, the cells were transfected using TransIT-LT1 (Mirus Bio. LLC; #MIR2305) with appropriate plasmids and then cultured in DMEM (Gibco; #11965118) containing 10% (v/v) FBS (Wisent; #080–150). Twenty-four hours after transfection, the transfected COS7 cells were washed with extracellular solution (ECS) containing 168 mM NaCl, 2.4 mM KCl, 20 mM HEPES, pH 7.4, 10 mM D-glucose, 2 mM CaCl_2_, 1.3 mM MgCl_2_, and 100 μg/ml bovine serum albumin (BSA; Sigma, #A9647); the cells were then incubated for 1 hour at 4 °C with NGL-3-Fc or SALM5-Fc (R&D Systems; #9385-SA) proteins diluted in ECS to 2.5 and 0.2 μM respectively. Cells were washed with ECS and subsequently fixed in prewarmed 4% (v/v) paraformaldehyde (PFA)/4% (w/v) sucrose in PBS for 12 minutes and blocked in 5% (v/v) normal donkey serum (NDS) and 3% (w/v) BSA in PBS for 1 hour at room temperature. COS7 cells were incubated with anti-LAR (0.1 μg/ml; goat; R&D Systems; #AF3004) or anti-HA (0.5 μg/ml; rabbit; Abcam; #ab9110) without permeabilization overnight at 4 °C. Cells were then incubated with highly cross-adsorbed Alexa dye–conjugated secondary antibodies generated in donkey toward the appropriate species (1.5 μg/ml; Jackson ImmunoResearch or ThermoFisher). Images were acquired on a Leica DM6 fluorescence microscope with a 40× 1.25 NA oil objective and a Hamamatsu C11440 ORCA-Flash 4.0 camera using LasX software (Leica). Images were acquired as 16-bit grayscale. For quantification, sets of cells were stained simultaneously and imaged with identical settings.

### Artificial synapse formation assay

All animal experiments were carried out in accordance with Canadian Council on Animal Care guidelines and approved by the IRCM Animal Care Committee. Cocultures of rat hippocampal neurons with HEK293T cells were set up as previously [87, 88]. Briefly, hippocampal neurons from E18 rat embryos were cultured on poly-L-lysine-coated glass coverslips in neurobasal medium (Gibco; #21103–049) supplemented with NeuroCult SM1 (StemCell; #05711) and GlutaMaX (Gibco; #35050061). For neuron-HEK293T coculture assays, appropriate plasmids were transfected in HEK293T cells using TransIT-LT1 (Mirus Bio. LLC; catalog number: MIR2305) and cultured in DMEM containing 10% (v/v) FBS. Twenty-four hours after transfection, cells were harvested by trypsinization and seeded on the neuron cultures at 14 days *in vitro* (DIV). After 24 hours of coculture, cells were fixed in prewarmed 4% (v/v) PFA/4% (w/v) sucrose in PBS for 12 min and blocked with 5% (v/v) NDS and 3% (w/v) BSA in PBS for 1 hr at room temperature. Cells were incubated with anti-LAR (0.1 μg/ml; goat; R&D Systems; #AF3004) or anti-HA (0.5 μg/ml; rabbit; Abcam; #ab9110) overnight at 4 °C to label transfected surface-expressed proteins. Cells were then permeabilized in 0.2% (v/v) Triton X-100 in PBS and incubated with anti-PSD95 (3.3 μg/ml; mouse; ThermoFisher, #MA1–045) overnight at 4 °C. Cells were then incubated with highly cross-adsorbed Alexa dye–conjugated secondary antibodies generated in donkey toward the appropriate species (1.5 μg/ml; Jackson ImmunoResearch or ThermoFisher). Images were acquired on a Leica DM6 fluorescence microscope with a 63× 1.40 numerical aperture (NA) oil objective and a Hamamatsu C11440 ORCA-Flash 4.0 camera using LasX software (Leica). Images were acquired as 16-bit grayscale. For quantification, sets of cells were stained simultaneously and imaged with identical settings.

### Image analysis

To quantify binding levels and cell surface expression levels, we measured the average intensity of each channel within the delineated COS7 or HEK293T cell area subtracted by the average intensity of the off-cell background. For *in situ* binding assays, the average intensity of bound soluble NGL-3-Fc protein was normalized using the average surface intensity of the LAR or CD4 protein signal. COS7 cells expressing similar levels of LAR or CD4 proteins were selected to quantify bound soluble NGL-3-Fc protein. Analyses were performed using Volocity 6.0, Excel for Microsoft 365 (Microsoft), and Prism 9 (GraphPad Software, Inc). For the artificial synapse formation assays, HEK293T cells displaying similar surface levels of LAR or CD4 proteins were imaged without considering the channel corresponding to PSD95 signal. To assess glutamatergic postsynaptic differentiation, the fluorescence channel corresponding to PSD95 was thresholded, and the total tresholded intensity within regions positive for surface LAR or HA was measured and normalized to HEK293T cells area. Analysis was performed using Metamorph 7.8 (Molecular Devices), Excel for Microsoft 365, and Prism 9. For the cellular aggregation assays, the aggregation index was calculated by dividing the number of particles with an area higher that of a single cell by the total number of particles. Analysis was performed using the Analyze Particles function of the FIJI processing package (ImageJ). Agostino’s K-squared test was used to assess normal distributions, and Bartlett’s test was used to check whether standard deviations (SDs) were significantly different across groups. To compare three or more groups with normal distribution, Welch’s ANOVA with Dunnett’s T3 post hoc analysis was used. For comparisons between three or more groups without normal distribution, Kruskal Wallis tests with Dunn’s post hoc analysis were used. Statistical significance was examined with appropriate tests as indicated in the figure legends. All data are reported as the mean ± SEM from at least three independent experiments, and statistical significance was defined as *p* < 0.05.

### Small Angle X-ray Scattering

Purified LAR variants are concentrated to different series (1 mg/ml, 1.5 mg/ml, 2 mg/ml and 2.5 mg/ml) in gel filtration buffer (150 mM NaCl, 100 mM Tris pH 7.5). SAXS data were collected at in-house instrument at iNANO at Aarhus University [89] at 20°C for 1800 second measurement for each sample. Data collection methodology is detailly described in [89]. Raw data processing, water scattering, background and beam-stop shadow corrections, data reduction and buffer subtraction are done by in-house software package Supersaxs (Oliveira & Pedersen, unpublished). The resulting data are subjected to Guinier fit analysis, Kratky analysis and IFT(GNOM) by using BioXTAS RAW software package [90]. Scattering curves are normalized to concentration and plotted in log-log scale with the associated error bars. Ab-initio models are determined by dummy residue modelling that are constructed from scattering profiles by using DAMMIF which is part of the ATSAS package and scattering data are fitted to models by using CRYSOL in ATSAS software package [91]. All the structural models are prepared by using The PyMOL Molecular Graphics System, Version 3.0 Schrödinger, LLC.

### Adapted Lipocyte Profiling

This assay is an adaptation of LipocyteProfiler and Cell Painting [55, 56]. U2OS cells were maintained in McCoy’s 5A medium supplemented with 10 % FBS and were plated in 384 well PhenoPlates (PerkinElmer # 6057302) at a density of 2K cells / well. After 24 hours, the medium was changed to McCoy’s 5A without FBS and the cells were transfected with GFP, LAR WT or missense variants using Lipofectamine^™^ 3000 Transfection Reagent (Invitrogen L3000015). To minimize plate-layout effects, each replicate of the different transfections (n=6) was placed as suggested from: https://carpenter-singh-lab.broadinstitute.org/blog/how-normalize-cell-painting-data. 24 hours after the transfection, the medium was supplemented with MitoTracker Deep Red (Invitrogen #M22426) to a final concentration of 500 nM and incubated for 30 min at 37 °C. Then all wells were subjected to a 16 % PFA solution to a final concentration of 4 % and kept for 20 min protected from light. This solution was then removed, and the wells were washed twice with 1x HBSS (Gibco #14025076) and incubated with a multi-stain solution containing 1 unit/mL Alexa Fluor^™^ 568 Phalloidin (Invitrogen A12380), 10 μg/mL Hoechst 33342 (Invitrogen H3570), 1.5 μg/mL Wheat Germ Agglutinin Alexa Fluor^™^ 555 Conjugate (Invitrogen W32464), 0.1 % Triton X-100 (Sigma Aldrich #X100) in 1x HBSS containing 1 % BSA and incubated for 30 min at RT protected from light. The solution was removed, wells washed twice in 1x HBSS and anti-LAR (R&D, AF3004) in 1x HBSS containing 1 % BSA was added to the wells and left ON at 4 °C. On the next day, the wells were washed twice with 1x HBSS and secondary stained with an Alexa Fluor^™^ 488 conjugated antibody (Sigma Aldrich # A-11055 for 2 hours at RT protected from light. Finally, the wells were washed three times with 1x HBSS and imaged using the Opera Phenix High Content screening system in confocal mode with the 20x objective and numerical aperture of 1.

Feature extraction and quantification was performed using CellProfiler. Prior to image processing, the images were flat field illumination correction was performed using mean averaging across all image sets, after which median smoothing was applied and the resulting image from the illumination correction function is then used to divide from the original image. Nuclei were identified with the Hoechst stain and then used to identify single cells through the WGA/Phalloidin stain. Cytoplasmic regions were found by subtracting the nuclei segments from the whole cell segments. For each of these compartments, measures related to size, shape, intensity, granularity, texture, colocalization and distance to neighboring objects were extracted. The data was normalized across the plate using subtraction of the median of each variable and dividing it with the interquartile range. α-LAR signal was used to identify transfected cells and only aggregate these for downstream analysis. Subsequent manual filtering steps included total LAR positive cell count >25 and removal of artefacts. The features were normalized across the plate and pr well means were calculated for each feature and used to generate per-feature Z-scores and p-values for each LAR variant. This normalized, filtered data was analyzed using R4.3.1.

Of note, the U2OS cells were used for their compatibility with the high-throughput format and easy transfection and growth in imaging plates. Analysis was performed directly between variants and WT, since several studies have shown that finding relevant negative controls in these highly sensitive morphological studies [71, 92].

All code used in the manuscript will be made available.
